# Distributions of CHN compounds in meteorites record organic syntheses in the early solar system

**DOI:** 10.1038/s41598-023-33595-0

**Published:** 2023-04-24

**Authors:** Yoshihiro Furukawa, Daisuke Saigusa, Kuniyuki Kano, Akira Uruno, Ritsumi Saito, Motoo Ito, Megumi Matsumoto, Junken Aoki, Masayuki Yamamoto, Tomoki Nakamura

**Affiliations:** 1 Department of Earth Science, Tohoku University, Sendai, Japan; 2 Laboratory of Biomedical and Analytical Sciences, Faculty of Pharma-Science, Teikyo University, Tokyo, Japan; 3 Department of Integrative Genomics, Tohoku Medical Megabank Organization, Tohoku University, Sendai, Japan; 4 Department of Health Chemistry, Graduate School of Pharmaceutical Sciences, The University of Tokyo, Tokyo, Japan; 5 Department of Medical Biochemistry, Graduate School of Medicine, Tohoku University, Sendai, Japan; 6 Kochi Institute for Core Sample Research, X-star, Japan Agency for Marine-Earth Science and Technology, Nankoku, Japan

**Keywords:** Asteroids, comets and Kuiper belt, Astrobiology, Meteoritics, Imaging studies, Mass spectrometry

## Abstract

Carbonaceous meteorites contain diverse soluble organic compounds. These compounds formed in the early solar system from volatiles accreted on tiny dust particles. However, the difference in the organic synthesis on respective dust particles in the early solar system remains unclear. We found micrometer-scale heterogeneous distributions of diverse CHN_1-2_ and CHN_1-2_O compounds in two primitive meteorites: the Murchison and NWA 801, using a surface-assisted laser desorption/ionization system connected to a high mass resolution mass spectrometer. These compounds contained mutual relationships of ± H_2_, ± CH_2_, ± H_2_O, and ± CH_2_O and showed highly similar distributions, indicating that they are the products of series reactions. The heterogeneity was caused by the micro-scale difference in the abundance of these compounds and the extent of the series reactions, indicating that these compounds formed on respective dust particles before asteroid accretion. The results of the present study provide evidence of heterogeneous volatile compositions and the extent of organic reactions among the dust particles that formed carbonaceous asteroids. The compositions of diverse small organic compounds associated with respective dust particles in meteorites are useful to understand different histories of volatile evolution in the early solar system.

## Introduction

Many primitive carbonaceous meteorites contain diverse organic compounds that formed and matured in the early solar system^1–9^. These organic compounds have been intensively investigated as primitive samples of the early solar system and organic compounds delivered to prebiotic earth^1,2,5–11^. Target-specific investigations of soluble compounds have demonstrated that primitive meteorites contain many small organic compounds such as amino acids, hydrocarbons, polyols, nucleobases, and carboxylic acids^1,5,7,9^. Based on these findings and laboratory investigations, many types of formation reactions have been proposed, including the Fischer–Tropsch type reaction for molecules with hydrocarbon chains, formose-type reaction for sugars, amino acids, amines, and carboxylic acids, and Strecker synthesis, and Michael addition for amino acids^7,10,12–17^. In addition to these named reactions, many of these compounds were proposed to have formed from reactions between simple compounds such as methanol, water, and ammonia by photochemical reactions^5,18–20^. Refractory organic matter, including solvent insoluble organic matter (IOM), is large complex carbonaceous matter and the most abundant type of organic matter in meteorites^1,2,5^. Several reactions, including the Fischer–Tropsch type and formose-type, have been discussed as their synthetic reactions^12,15,17,21,22^. More recently, diverse mass signals detected from extracts of primitive meteorites by non-target high-resolution mass spectrometry revealed that such primitive meteorites contain huge variations of small soluble organic compounds (i.e. 50,000 elemental formula and potentially several millions of compounds)^3,4,8,23^. The characteristics of the diverse small organic compounds should provide meaningful information to understand the dominant formation reactions of the organic matter.

Carbonaceous meteorites contain micrometer-sized mineral particles that accreted to form parent asteroids of meteorites^24^. Thus, the organic compounds associated with the tiny particles may record distinct reactions that created the organic compounds in the early solar system. Refractory organic matter in carbonaceous chondrites has been characterized in-situ using a range of analytical techniques, including scanning transmission electron microscope equipped with electron energy loss spectroscopy (STEM-EELS), scanning transmission X-ray microscopy (STXM), infrared spectroscopy (IR), and secondary ion mass spectrometry (SIMS)^25–29^. Micrometer-scale heterogeneity of chemical bonds and stable isotopes in the carbonaceous matter in extraterrestrial samples indicates the diversity of organic matter associated with mineral particles^25–30^. Conversely, small organic compounds have been investigated in solvent extracts from bulk meteorite chips^3–10,23,31,32^. Thus, distributions of soluble organic compounds were homogenized in these analyses. Distributions of diverse small organic compounds can provide substantial information for understating the formation processes of these organic compounds.

Organic compounds in small regions of extraterrestrial samples (e.g. ~5 µm) have been investigated with laser-desorption laser-ionization mass spectrometer, primarily for polycyclic aromatic hydrocarbons^33–35^. This previous analysis has a low mass resolution (e.g. larger than 5 ppm), which is insufficient to constrain the compositions of diverse organic compounds^34^. Spatial distributions of CHN compounds (i.e. possible alkyl N-heterocyclic compounds) have been reported using desorption electrospray ionization (DESI) coupled with high-resolution mass spectrometry (e.g. ~5 ppm)^36,37^. However, the spatial resolution of this previous analysis (i.e. ~200 µm) is insufficient to evaluate organic compounds associated with micrometer-sized mineral particles in meteorites. Thus, the compositions of diverse organic compounds associated with the micrometer-sized mineral particles are unknown due to analytical limitations. The present study uses a high spatial-resolution and high mass-resolution analysis for meteorites to characterize the difference between organic compounds associated with different dust particles in meteorites.

## Results

### Distributions of CHN and CHNO compounds in NWA 801 and the Murchison meteorite

**Figure 1:**
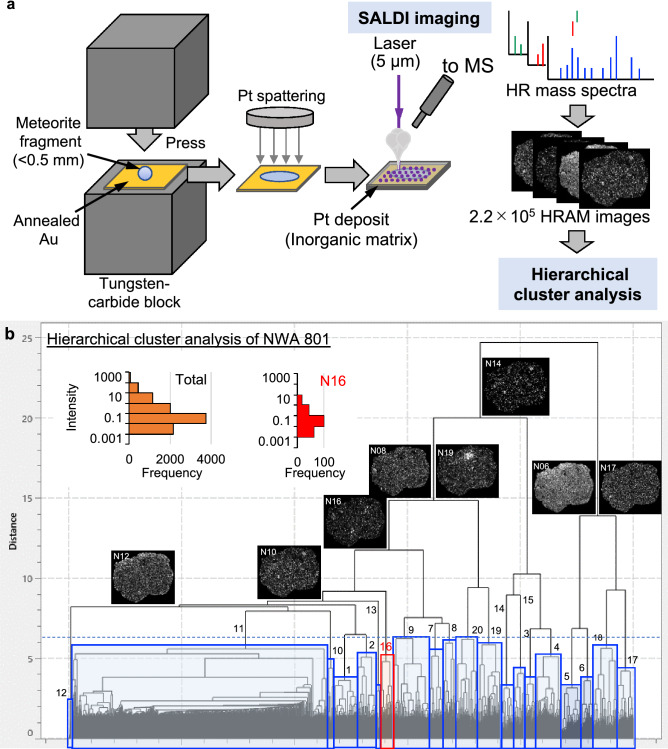
Pt-SALDI imaging and hierarchical cluster analysis of NWA 801 meteorite. (**a**) Pt-SALDI imaging of meteorite. Pt inorganic matrix was sputtered on a compressed meteorite fragment. The sample was analyzed by a SALDI system connected to a high mass resolution mass spectrometer. Distributions of 2.2 × 10^5^ signals were constructed using spot-by-spot high-resolution accurate mass (HRAM) signals. (**b**) Hierarchical cluster analysis of major signals detected in the NWA 801 meteorite. The similarity in the spatial distribution of signals was categorized into 20 clusters. The relative dissimilarity of spatial distributions between the clusters is represented by the distance of the horizontal bar between the clusters.

The distributions of organic compounds in micrometer resolution in two primitive carbonaceous meteorites were investigated using a surface-assisted laser desorption/ionization (SALDI) system connected to a high mass resolution mass spectrometer (spatial resolution of 5 µm and mass resolution of ~5 ppm; Fig. 1a). In this study, a platinum sputtering deposit that had previously been used for SALDI analyses of biological and industrial samples was used as the inorganic matrix^38,39^. The inner part of the NWA 801 and Murchison meteorites were used for the analysis. The NWA 801 meteorite investigated in this study is a carbonaceous chondrite of Renazzo-type (CR) and had a negligible aqueous alteration in its asteroid (see “Materials and methods”). The Murchison meteorite is a well-known carbonaceous chondrite of Mighei-type (CM) with a substantial aqueous alteration in the parent asteroid^40,41^. The investigated meteorite samples compressed on an Au substrate have approximately 1 mm^2^ area. Thus, they contain enough particles of several micrometers and even smaller to evaluate the meteorite matrix, although many meteorites and asteroids have some small-scale to large-scale heterogeneities.

**Figure 2:**
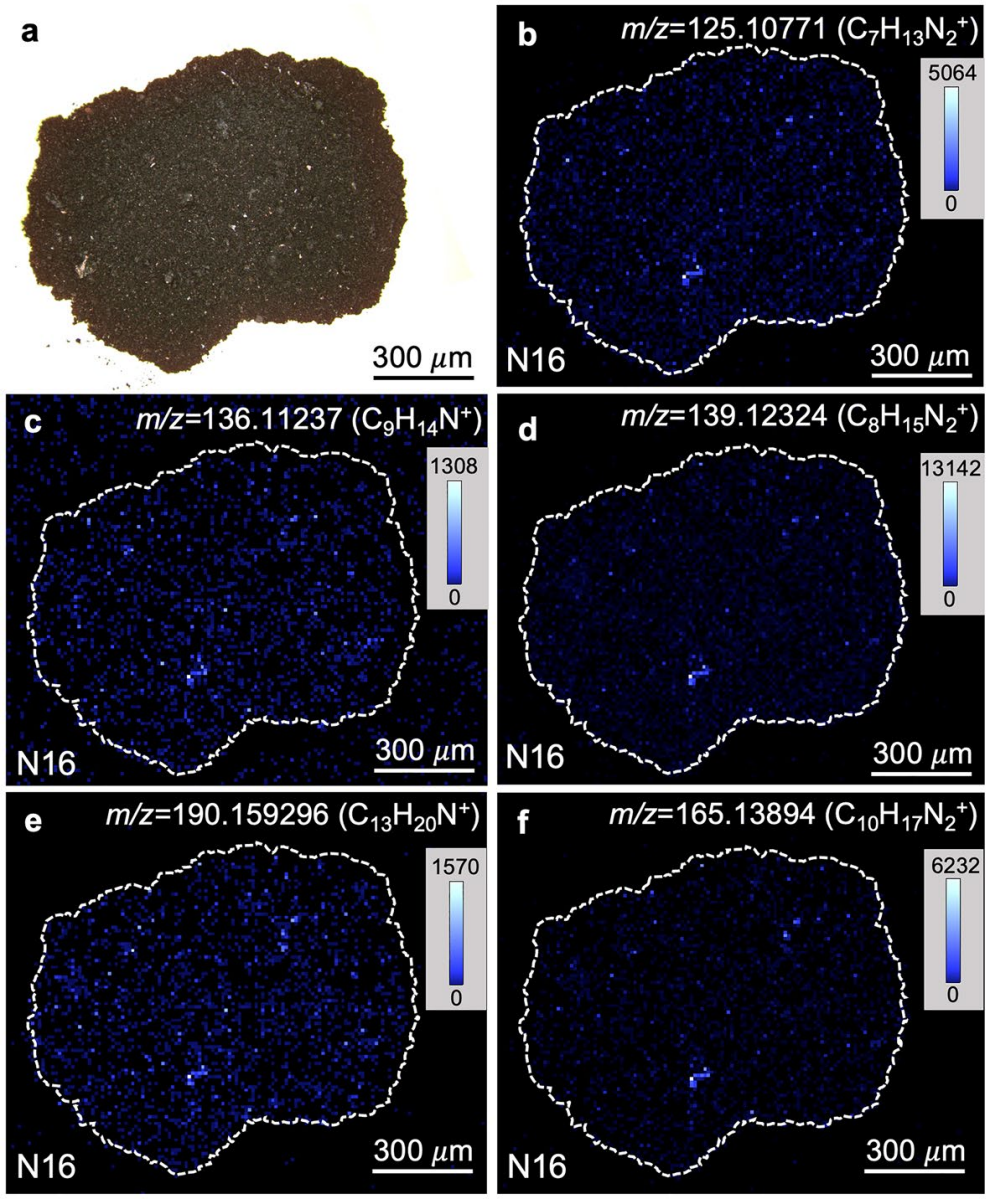
Similarity in the spatial distributions of CNH compounds in NWA 801 meteorite. (**a**) Optical reflection image. (**b**) Mass imaging of *m/z* = 125.10771 in cluster N16 corresponds to C_7_H_13_N_2_^+^. (**c**) Mass imaging of *m/z* = 136.11237 in cluster N16 corresponds to C_9_H_14_N^+^. (**d**) Mass imaging of *m/z* = 139.12324 in cluster N16 corresponds to C_8_H_15_N_2_^+^. (**e**) Mass imaging of *m/z* = 190.159296 in cluster N16 corresponds to C_13_H_20_N^+^. (**f**) Mass imaging of *m/z* = 165.13894 in cluster N16 corresponds to C_10_H_17_N_2_^+^.

**Figure 3:**
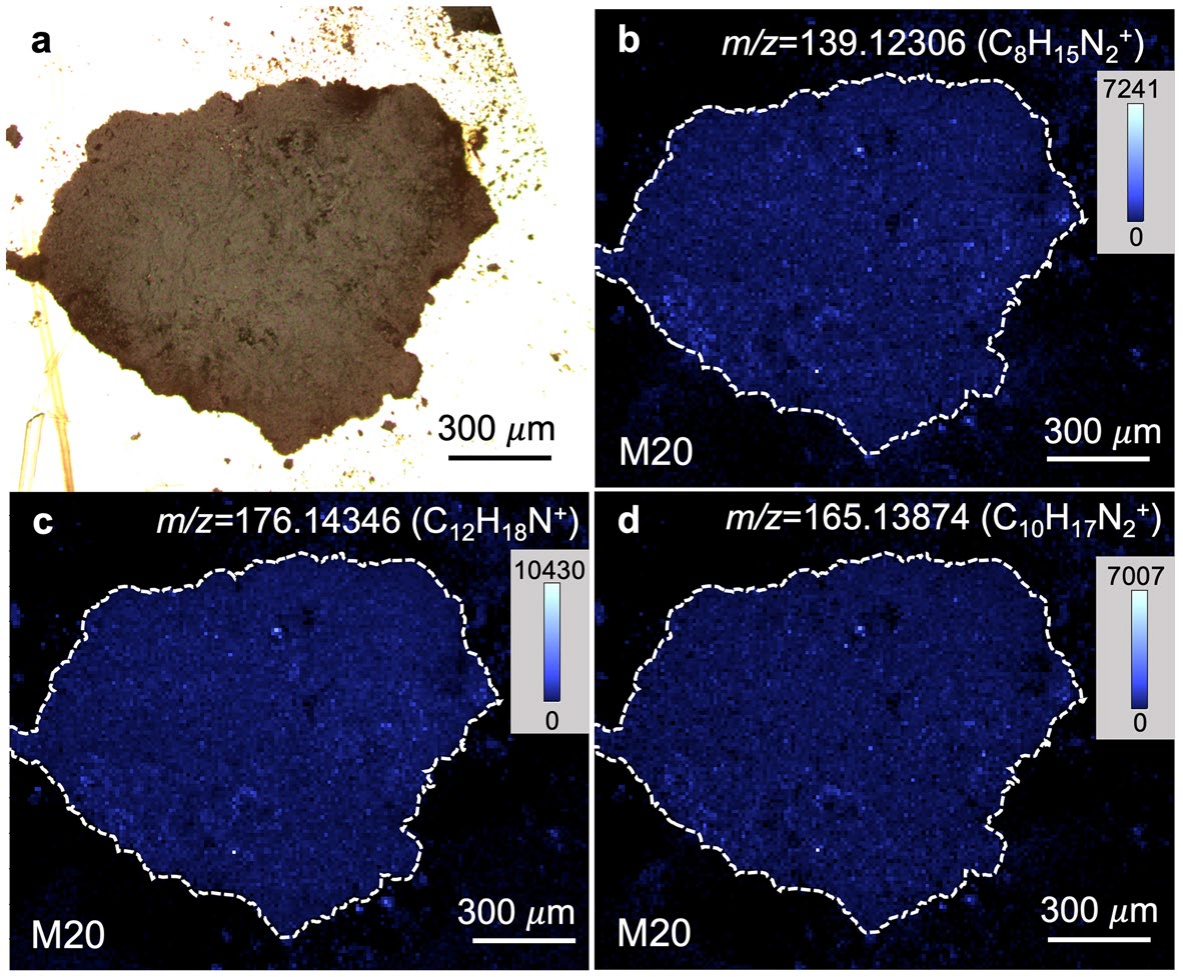
Similarity in the spatial distributions of CHN compounds in the Murchison meteorite. (**a**) Optical reflection image. (**b**) Mass imaging of *m/z* = 139.12306 in cluster M20 corresponds to C_8_H_15_N_2_^+^. (**c**) Mass imaging of *m/z* = 176.14346 in cluster M20 corresponds to C_12_H_18_N^+^. (**d**) Mass imaging of *m/z* = 165.13874 in cluster M20 corresponds to C_10_H_17_N_2_^+^.

**Figure 4:**
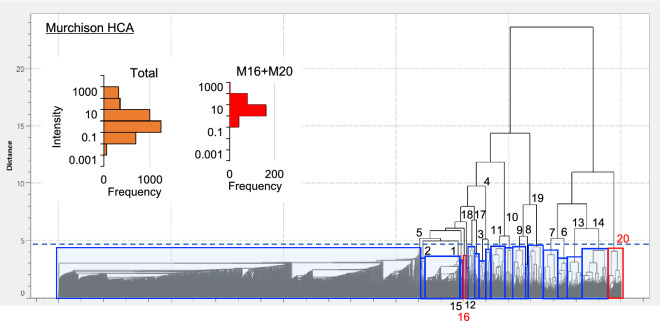
Hierarchical cluster analysis of major signals detected in the Murchison meteorite. The relative dissimilarity of spatial distributions between the clusters is represented by the distance of the horizontal bar between the clusters. The signals in clusters M16 and M20 correspond to the signals in cluster N16 of the NWA801 but are divided into M16 and M20 in this HCA.

**Figure 5:**
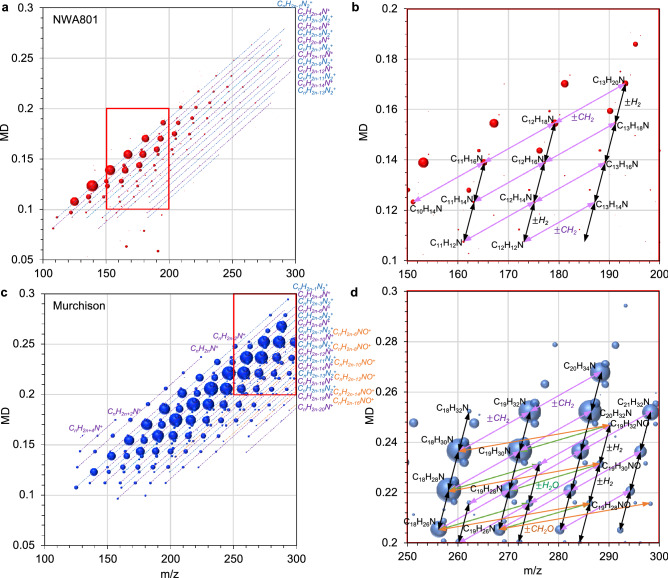
Mass-defect (MD) plots of mass signals in the clusters of N16 of NWA 801and M60 and M20 of the Murchison meteorite. Filled colored balloons represent detected m/z and their intensities. (**a**) Cluster N16. (**b**) Magnification of the red square in (**a**). (**c**) Cluster M16 and M20. (**d**) Magnification of the red square in **c**. The size of the balloons represents the signal intensities. MD = (exact mass) – (nominal mass).

**Figure 6:**
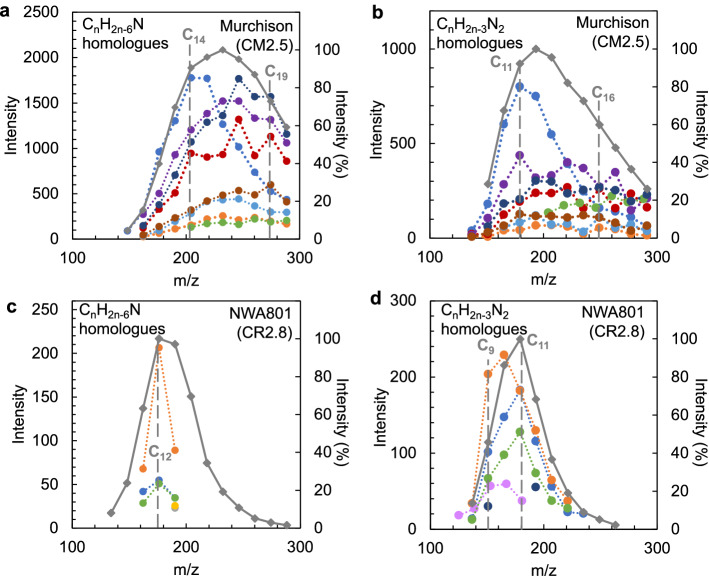
Intensities of the CHN compounds at different micro-spots and overall. (**a**) C_n_H_2n−6_N homologs in the Murchison meteorite. (**b**) C_n_H_2n−3_N_2_ homologs in the Murchison meteorite. (**c**) C_n_H_2n−6_N homologs in the NWA 801 meteorite. (**d**) C_n_H_2n−3_N_2_ homologs in the NWA 801 meteorite. Filled colored circles with dotted lines represent the profile from micro-spots. Filled diamonds with solid gray lines represent the profile from the entire region of the samples, shown in percentage intensities.

The spatial distributions of the major 10,000 mass signals of the detected ~220,000 mass signals (*m/z* 100 to 300) were categorized into 20 clusters based on their similarity using hierarchical cluster analysis (HCA) (Figs. 1b and 2, 3 and 4). This distribution analysis has been used for biological samples and was applied to the astronomical samples in this study^42^. Mass signals in many clusters could not be assigned, however, several clusters contained diverse mass signals of CHN, CHNO, and CHO compounds. Cluster N16 of NWA 801 and M16 and M20 of the Murchison meteorite were composed of diverse CHN and CHNO compounds with the elemental formula of C_n_H_2n-m_N_1-2_O_0-1_ (Table S1 and Fig. 5). This clearly indicates that these molecules have similar spatial distributions (Figs. 2 and 3). These mass signals have mutual relations of ± CH_2_, ± H_2_O, ± H_2_, and ± CH_2_O (Fig. 5). The distributions of this type of compound were significantly heterogeneous in both meteorites (Figs. 2 and 3). Spot-by-spot analysis showed different carbon abundance of the CHN compounds in the different spots of the Murchison meteorite (Fig. 6). The carbon abundance of these compounds exhibited more differences between the NWA 801 meteorite and the Murchison meteorite (i.e. higher in the Murchison than NWA 801) than the difference between varying spots in the Murchison meteorite (Fig. 5a and 5c). The difference between the CM and CR chondrite was also observed in a previous bulk analysis of meteorite extracts from the Murchison (CM) and Y-002540 (CR)^6^.

### Stable nitrogen isotope compositions in the CHN-rich spot in NWA 801

**Figure 7:**
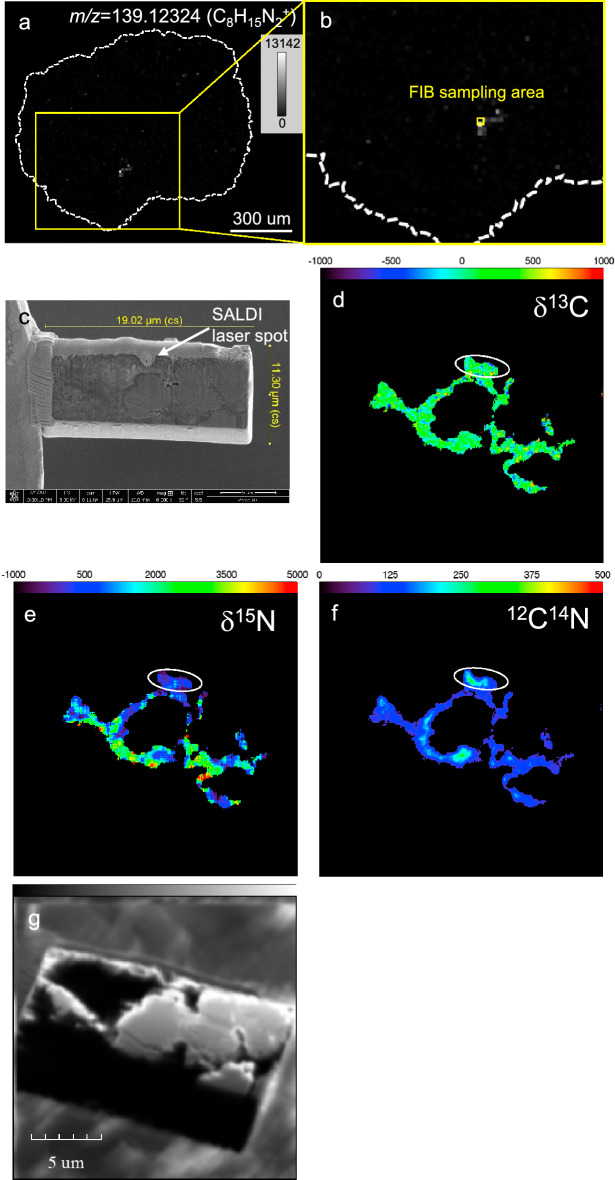
NanoSIMS analysis of the FIB section collected from an area of concentrated C–H–N compounds in the NWA 801 sample. **(a**,**b)** Sampling area on the distribution of C_n_H_2n-1_N_2_ compound. (**c**) SEM image of FIB section from the area shown in (**a**). (**d**) δ^13^C map of the FIB section. (**e**) δ^15^N map of the FIB section. The circled region has δ^15^N = +292 ± 43‰. (**f**) ^12^C^14^N image of the FIB section. (**g**) NanoSIMS secondary electron image of the FIB section. The organic-rich regions have been selected using distributions of ^12^C ions in the FIB section applying a 10% threshold of the total ^12^C ion counts. Organic-rich regions, therefore, were distinguished with a spatial resolution of ~100 nm.

The most concentrated area of these CHN compounds was analyzed using nanoscale secondary ion mass spectrometry (NanoSIMS). The NanoSIMS isotope analysis demonstrated a ^15^N enrichment in this area, which was δ^15^N = + 292 ± 43 ‰ (Fig. 7). The distribution of the above compounds did not exhibit clear relationships with the distribution of major elements (i.e. Mg, Fe, Al and S) detected in a scanning electron microscope (SEM) equipped with an energy-dispersive X-ray spectrometer (SEM-EDS) (Fig. S1).

## Discussion

### Evaluation of potential terrestrial contamination

Terrestrial contamination of biological organic compounds is an important issue in the analyses of organic matter in meteorites. However, most of the CHN and CHNO compounds are not biological compounds and the sequential molecular pattern (i.e. detected molecules and their abundances) is significantly different from biological compounds that are formed selectively by enzymatic activity and cellular reactions, although there are many types of biological organic matter and thus it is difficult to be compared with all the molecular trends of biological/terrestrial organic matter in this manuscript (Fig. 5). Previous petrological descriptions of a piece of the NWA 801 meteorite reported that it was moderately or extensively weathered on Earth^43^. However, in the piece of meteorite analyzed in the present study, the extent of mineral weathering was minor, with a small amount of iron hydroxide in the matrix (see “Materials and methods”). This is most likely because the outer surface of this meteorite is entirely covered by glassy fusion crust. The sugar molecules extracted from the same piece of this meteorite have completely different carbon isotope compositions from terrestrial sugars^7^. Further, when terrestrial fluids containing biologically related compounds migrate in meteorites, these compounds should be distributed either homogeneously over exposed surfaces and within the meteorites or locally along cracks in the meteorites. In this study, the CHN and CHNO compounds were heterogeneously distributed in micrometer-scale (Figs. 2 and 3). This distribution cannot be reproduced by contamination of terrestrial fluids containing the organic compounds. Further, the authigenic mass signals in the meteorite were clearly distinguished from small amounts of contaminant mass signals detected in the outer region of the meteorite (Figs. S2–S4). Therefore, the CHN and CHNO compounds are not originated from terrestrial contamination but were formed in non-biological reactions in the early solar system.

### The synthetic reactions and environments of the diverse CHN and CHNO compounds

The C_n_H_2n-m_N_1-2_ signals have also been detected from the extracts of several meteorites by Fourier transform ion cyclotron resonance mass spectrometry (FTICRMS), and liquid chromatography mass spectrometry (LC/MS) as one of the major signal types and assigned as alkyl N-heterocyclic compounds such as alkyl pyridines and alkyl imidazoles^3,6,8,32^. The C_n_H_2n-m_N_1-2_ compounds found in the present study are also likely to be alkyl N-heterocyclic compounds because they have the same molecular compositions as previous studies. The C_n_H_2n-m_N_1-2_O compounds would be N-heterocyclic compounds with a hydroxyl group.

Diverse CHN compounds, CHNO compounds, CHO compounds, sugars, and sugar-related compounds form in low-temperature laboratory simulations of photochemical reactions of interstellar and cometary ice analogues using simple and abundant observed molecules, such as water, ammonia, and methanol^18,19,44^. Various CHN compounds can also be formed in laboratory hydrothermal decomposition of hexamethylenetetramine (HMT), which is the major product of such low-temperature laboratory simulations of photochemical reactions of interstellar and cometary ice analogues and found recently in meteorites^45–49^. Several CHN compounds can also be formed by simple heating of formaldehyde and ammonia in alkaline solutions^50^. Formaldehyde (CH_2_O) was detected in many primitive meteorites, comets, and star-forming regions^51–54^. Ammonia has been found in meteorites and comets and observed in proto-planetary systems^55–57^. Thus, the synthetic reactions of the CHN compounds were possible both photochemically before asteroid accretion and thermochemically in asteroids.

The mutual relations of ± CH_2_ between a part of the detected mass signals is generally consistent with the contributions of a Fischer–Tropsch type reaction. However, previous studies discuss the formation of alkyl pyridines by oligomerization of aldehyde, followed by a Chichibabin pyridine synthesis, including reductive amination, from aldehydes and ammonia in solution. The mutual relations of ± CH_2_O identified in this study are consistent with the oligomerization of aldehyde. The substantial mutual relations of ± CH_2_ can be created by aldehyde oligomerization (+CH_2_O) followed by their substantial dehydration (–H_2_O) and varying degree of hydrogenation of the unsaturated compounds (–H_2_) (Fig. 5d and Fig. S5). Further, oligomerization of aldehyde could be promoted photochemically before asteroid accretion^58,59^, although it is unclear whether all these reactions progress photochemically in ice. Therefore, the reaction sequence of oligomerization of aldehyde, followed by a Chichibabin pyridine synthesis, hydrogenation and dehydration is more reasonably explain all the mutual relations between the detected mass signals. In this case, relatively small abundances of ± CH_2_O relations indicate substantial dehydration that converted most of the CHNO into CHN (Fig. S5). The oligomerization of aldehyde is the basic reaction of the formose reaction in which diverse lengths of sugars and sugar-related compounds form^60^. The formose reaction is also known to form many sugar-related compounds, including sugar alcohols and sugar acids, and diverse CHO compounds^61,62^. Reactions between aldehydes and ammonia are known to form IOM-like matter with carboxylic acids, amines, sugars, and amino acids^15,17,21^. The amino acids and IOM-analogue have similar relative carbon isotopic compositions to meteorites (i.e. lower δ^13^C values of IOM than amino acids) through the formation of the amino acids with the remaining aldehydes of the formose reaction^17^. Thus, the formation of diverse CHN compounds is consistent with the formation reactions of other organic compounds, given that they formed via a formose-type reaction.

The similar spatial distributions of diverse CHN and CHNO compounds identified in this study differ from the distributions found in a previous analysis by DESI-MS in the Murchison meteorite, whereby different CHN and CHNO compounds had different spatial distributions^36,37^. The varying characteristics in distribution were most likely due to the difference in organic compounds that can be ionized by DESI and SALDI. DESI-MS detects organic compounds extracted by a micro-spray, while SALDI-MS detects organic compounds that are desorbed and ionized by laser ablation. Thus, DESI imaging would show the distributions of highly mobile organic compounds, such as organic compounds formed by aqueous processes in asteroids. In contrast, SALDI imaging would also show the distributions of poorly mobile organic compounds, such as organic compounds surrounded by insoluble organic matter and minerals. Previous studies reported that IOM release diverse organic compounds including CHN compounds by laser ablation^63,64^. Given that the CHN compounds detected in the present study are protonated ions of the alkyl N-heterocycles, they are different from the fragments of IOM. It is unlikely that the distributions of CNH signals shown in the present study reflect the distributions of IOM because carbon distributes far more homogeneously in the matrix of NWA 801 (Fig. 7d). Given this and the superior spatial resolution, SALDI imaging is more useful to investigate the spatial distributions of organic compounds authigenic to micro-particles in meteorites.

The micrometer-scale heterogeneous spatial distributions of diverse CHN and CHNO compounds found in the present study demonstrate that the majority of these compounds remained on these particles after the aqueous processes. Further, it indicates that these compounds formed by series reactions on the particles before asteroid accretion and/or after asteroid accretion without micrometer-scale diffusion during the aqueous processes. Most tiny mineral particles in asteroids formed in the early solar system and accreted to form asteroids^24,65^. Volatiles such as water, methanol, formaldehyde, and ammonia are regarded to be distributed as ice on the mineral particles in molecular cloud and the outer side of the proto-solar disk depending on their freezing temperatures and their abundances^53,66^. Photochemical and thermochemical reactions were not ubiquitous but rather limited in the molecular cloud and the surface of the proto-solar disk^66^. The difference in the organic compositions associated with different dust particles in these meteorites can occur because of the variation in volatile compositions on the particles and/or the difference in photochemical/thermochemical processes before the asteroid accretion. Spot-by-spot mass spectra of the CHN compounds show that the profiles of the molecular weight (i.e. the length distribution of the hydrocarbon chain) in the CHN compounds are different in different spot (Fig. 6). This suggests that the heterogeneous distributions were caused by the difference in the reaction extent of aldol addition such as shown in Fig. S5 as well as the difference in the abundance of these CHN compounds on different particles. This indicates that the difference was initially caused before the asteroidal aqueous processes. The asteroidal aqueous processes most likely progressed the reactions in the parent asteroid of the Murchison meteorite, whereas the extent was rather limited in that of NWA 801. The insoluble organic matter associated with these compounds would have contributed to the preservation of the micrometer-scale heterogeneous distributions in the aqueously altered meteorites by limiting the diffusion of water soluble CHN compounds. The diversity in the volatile compositions and the reaction extent among different dust particles as well as the formation of small organic compounds before asteroid accretion have been discussed based on laboratory and theoretical simulations, cometary volatiles, and telescope observations^18–20,45,53,66^. The different extents of aldol addition to form CHN compounds recorded in different spots support these discussions and further provides the evidence on the reaction process based on the information from actual meteorites.

The spot-by-spot difference in the compositions of the CHN compounds and their abundances could have been provided by the mixing of the dust particles that have different histories before their parent body accretion. Presolar molecular cloud and outer solar system of proto-solar disk would be the places where reactions could occur under extremely cold conditions. Distributions of meteorite organic matter formed in such environments have been discussed based on its hydrogen and nitrogen isotope anomalies using NanoSIMS^26–29,67^. The CR meteorite is known to contain more abundant ^15^N-enriched refractory organic matter than CM meteorites^2^. ^15^N-enrichment is discussed as a characteristic of N-bearing compounds that originated from the extremely cold presolar molecular cloud or outer solar system because nitrogen isotope fractionation occurs at extremely cold environments^26,27^. The ^15^N enrichment in the area of the concentrated CHN compounds in NWA 801 (δ^15^N = +292 ± 43‰) was high, however it was much lower than the extremely ^15^N-enriched organic globules found in the meteorite (δ^15^N = +2,200 ± 320)^29^. The δ^15^N value found at the CHN-concentrated area was also lower than N-bearing compounds observed from comets whereas the value is in the range of interstellar HCN which have wide range of ^15^N enrichment^68^. This suggests that the nitrogen-bearing compounds that were used to form the CHN compounds in this region was the remaining fraction of the interstellar nitrogen-bearing compounds or the mixture of inner and outer solar nitrogen-bearing compounds.

### Advantages of the high-resolution Pt-SALDI imaging and HCA of distributions

Pt-SALDI imaging by high-resolution MS enabled the acquisition of high spatial resolution images of diverse compounds in meteorites. HCA enabled the categorization of mass signals that have similar distributions. MD plots of the compounds visualized the reaction network that formed these diverse compounds. This series of analyses are useful for establishing the connection between mass signals of diverse compounds and their origins. Further, the sample preparation for Pt-SALDI is highly compatible with micrometer-scale mineral and isotope analyses of meteorites. Thus, we anticipate that the application of these coordinated analyses to many astronomical samples will provide a more comprehensive understanding of the origin and evolution of the early solar system.

## Materials and methods

The inner part of a commercially available NWA 801 and Murchison meteorite were used for the analysis. The NWA 801 meteorite was covered entirely by glassy fusion clast, which might have decreased the extent of terrestrial weathering. Synchrotron X-ray diffraction showed that the NWA 801 used in this study contained no detectable phyllosilicate and small amounts of iron hydroxide in the meteorite matrix^7^. Back-scattered electron images of mesostasis glass and kamacite in chondrules in this meteorite showed almost no aqueous alteration product^7^. Further, the solid-state NMR spectrum of insoluble organic matter isolated from this meteorite had substantially high aliphatic signals to aromatic signals, indicating limited levels of maturation of insoluble organic compounds^7^. This evidence on mineralogy and organic chemistry indicates that the NWA 801 meteorite used in this study experienced a negligible degree of aqueous processes in its parent asteroid and the limited extent of weathering on the earth^7^. The NWA 801 meteorite is known to be a breccia^69^. The fragment investigated in this study would have been a less aqueously altered section among the different lithologies.

Approximately 500-μm-fragments of these meteorites were pressed on an annealed gold sheet of 100 μm thick with two tungsten carbide blocks using a hydraulic press. Surfaces of the tungsten carbide blocks were polished to create flat surfaces with a roughness of < 1 μm. The surface roughness of the meteorite samples was analyzed using a laser microscope (OLS5000; OLYMPUS) and determined as less than 2.5 μm in 4600 μm^2^ (Fig. S6). Optical microscope images were taken after the gold sheet was placed on a glass slide. Then, Pt sputtering was applied to form a Pt sputtering layer (~20 nm) as the inorganic matrix. This matrix was used to avoid the contamination of organic compounds from matrix chemicals and has been used in various MALDI analyses as SALDI^39,70^.

Pt-SALDI high-resolution mass spectrometry (Pt-SALDI HRMS) imaging of samples was conducted with a MALDI laser system (AP-SMALDI5, TransMIT) connected to the Fourier transform orbital trapping MS (QExactive, Thermo Fisher Scientific) in positive ion mode with the mass range of 100 to 300 at a mass resolution of 140,000. The laser energy was optimized to obtain a laser spot size of 5 µm. The solid-state laser was operated at 60 Hz with 30 laser pulses applied per spot. The hierarchical cluster analysis was conducted using IMAGEREVEAL MS (Shimadzu).

Elemental distribution analysis was conducted after the Pt-SALDI imaging using a scanning electron microscope (JSM-7001F, JEOL) equipped with an energy-dispersive X-ray spectrometer (INCA x-act). Micro-sampling for SIMS analysis was conducted with a dual-beam focused ion beam (FIB) system (Quanta 3D 200i, Thermo Fisher Scientific). A region of interest was extracted as a thick slice (~5 µm) from the meteorite section and mounted on a Cu grid using the FIB system. In the FIB processing, we used a Ga^+^ ion beam at 30 kV and 0.1–20 nA. The damage layers formed on the slice during the processing were removed by a weak Ga^+^ ion beam at 5 kV and 30 pA.

We conducted hydrogen, carbon, and nitrogen isotope imaging analyses of the FIB section with the JAMSTEC NanoSIMS 50 L. Briefly, a focused Cs^+^ primary ion beam of 1.2 to 4 pA was emitted to the rastered areas of 20 × 20 μm^2^ on the sample with 1-hydroxybenzotriazole hydrate (HOBT, C_6_H_5_N_3_O·xH_2_O, calculated as x = 1) as the standard isotopic material. The spatial resolution was approximately 100 nm for C and N isotope images. Each run was repeatedly scanned 25–30 times over the same area. The images consisted of 256 × 256 pixels with an acquisition time of 10,000 μs/pixel (655.4 sec/frame). Each measurement was started after stabilizing the secondary ion intensities following a pre-sputtering procedure of approximately 3 min. The sample was coated with a thin (10 nm) film of Au to mitigate the electrostatic charge on the surface. During the analysis, the mass peaks were centered automatically every five cycles. The final isotope images were generated from regions that had statistically sufficient counts. Detailed measurement conditions and isotopic standards are described in a previous study^71^.

## Supplementary Information

Supplementary Information.

## Data Availability

The datasets used and/or analysed during the current study available from the corresponding author on reasonable request.
