# Supplementary material for: Distributions of CHN compounds in meteorites record organic syntheses in the early solar system
Source: Sci Rep. 2023 Apr 24;13:6683. doi: 10.1038/s41598-023-33595-0 (PMC10125961; doi:10.1038/s41598-023-33595-0)
Supplement: Supplementary file 1 — Supplementary Information. [file 41598_2023_33595_MOESM1_ESM.docx]

**Supplementary Information**

**Distributions of CHN compounds in meteorites record organic syntheses in the early solar system**

Yoshihiro Furukawa^1*^, Daisuke Saigusa^2,3^, Kuniyuki Kano^4^, Akira Uruno^3,5^, Ritsumi Saito^3,5^, Motoo Ito^6^, Megumi Matsumoto^1^, Junken Aoki^4^, Masayuki Yamamoto^3,5^, Tomoki Nakamura^1^

**Affiliations:**

^1^Department of Earth Science, Tohoku University

^2^Laboratory of Biomedical and Analytical Sciences, Faculty of Pharma-Science, Teikyo University.

^3^Department of Integrative Genomics, Tohoku Medical Megabank Organization, Tohoku University

^4^Department of Health Chemistry, Graduate School of Pharmaceutical Sciences, The University of Tokyo

^5^Department of Medical Biochemistry, Graduate School of Medicine, Tohoku University

^6^Kochi Institute for Core Sample Research, X-star, Japan Agency for Marine-Earth Science and Technology

*Correspondence to: Yoshihiro Furukawa (furukawa@tohoku.ac.jp)


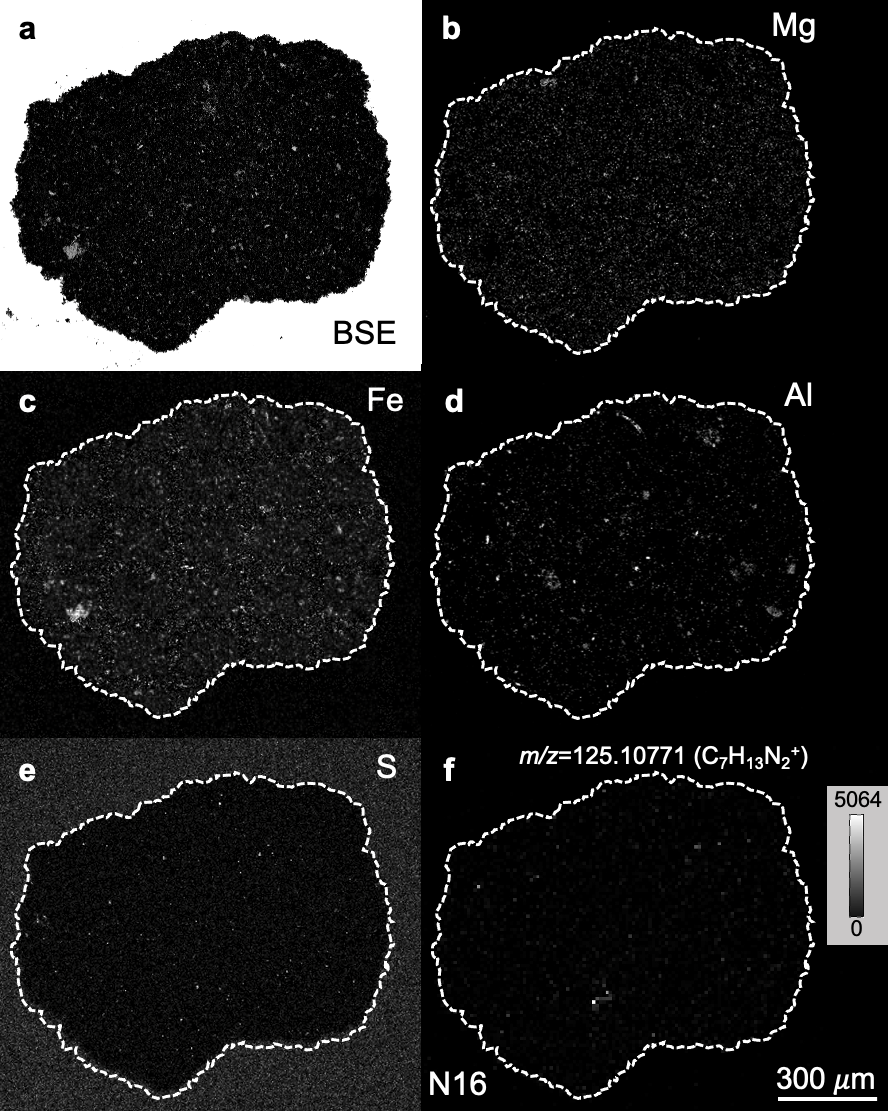


**Fig. S1. Elemental distributions on the NWA801 meteorite sample.** (**a**) Back scattered electron microscope image. Elemental distributions of Mg (**b**), Fe (**c**), Al (**d**), and S (**e**). (**f**) Distributions of mass signals of C_7_H_13_N_2_^+^ (m/z = 125.10771).

**Fig. S2. Mass spectra on the spots in the NWA801 meteorite sample.** (**a**) Mass distribution of *m/z* 525.5056. (**b**) Mass spectrum from spot #2. (**c**) Mass spectrum from spot #3.

**Fig. S3. Mass spectra on the spots in the NWA801 meteorite sample.** (**a**) Total signal image. (**b**) Mass spectrum from spot #1. (**c**) Mass spectrum from spot #2.

**Fig. S4. Mass spectra on the spots outside of the NWA801 meteorite sample.** (**a**) Total signal image. (**b**) Mass spectrum from spot #1. (**c**) Mass spectrum from spot #2.

**
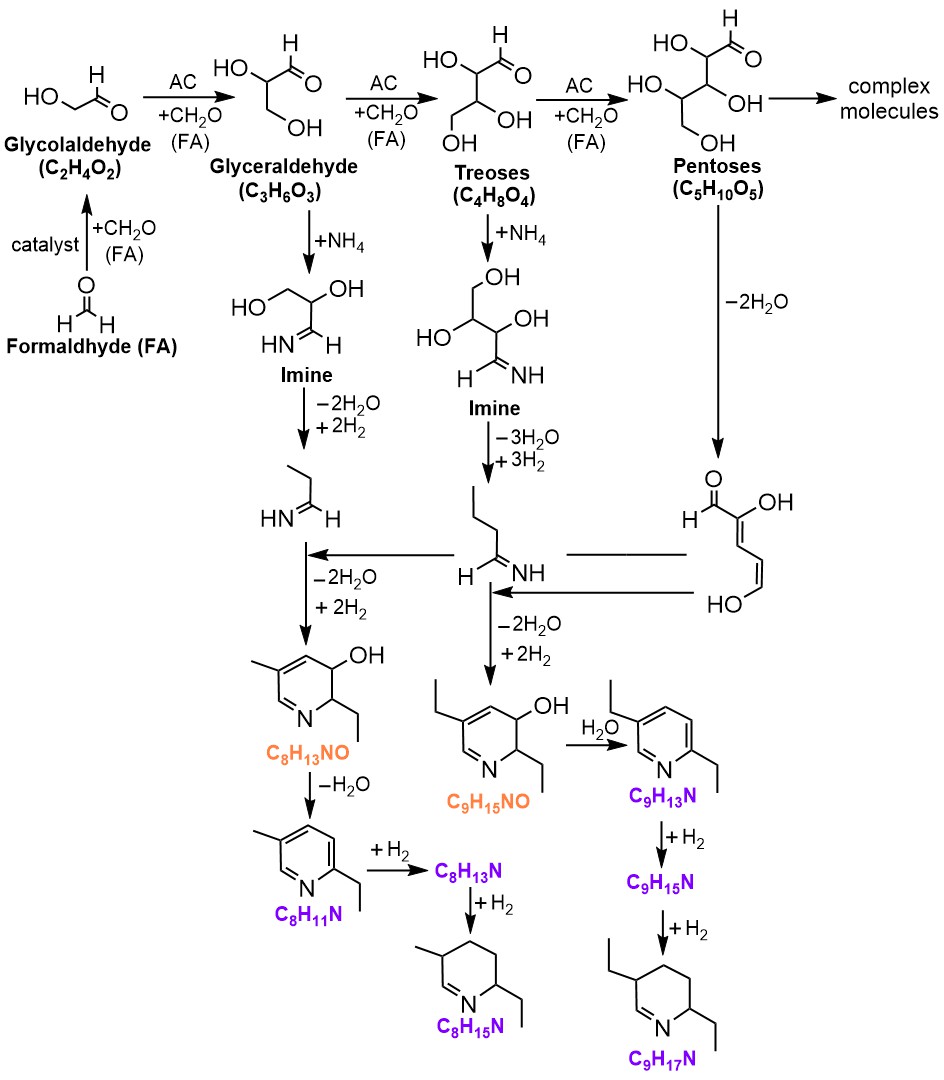
**

**Fig. S5. Possible reactions to form CHN_1–2_ and CHN_1–2_O compounds associated with a formose-type reaction and a Chichibabin pyridine synthesis.** AC represents aldol condensation. The compounds shown in this figure are examples of the possible compounds. Specific molecular structures are not investigated in this study, and the meteorites should contain many isomers.


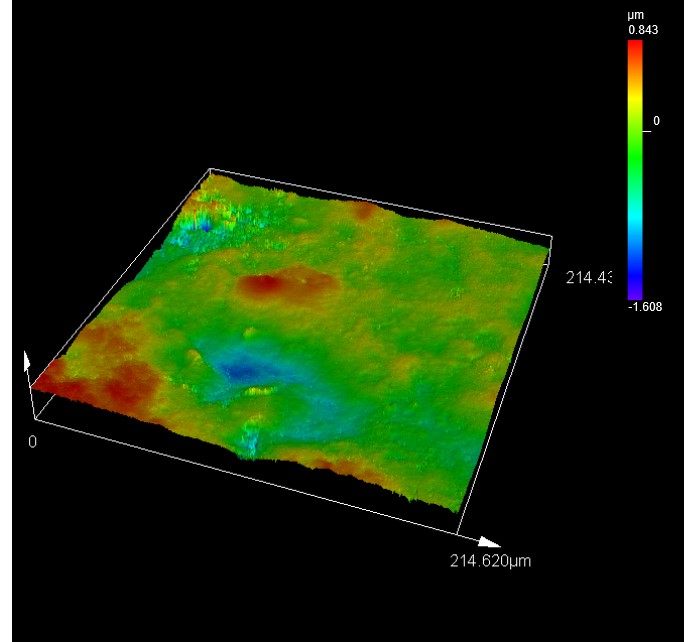


**Fig. S6. Surface flatness of the pressed sample of NWA 801.** The surface roughness is less than 2.5 μm in 4,600 μm^2^.

**Table S1.** Measured m/z from NWA801 and calculated m/z of the assigned C_n_H_2n-1_N_2_^+^ compounds.
